# Impact of Flap Size and Comorbidities on Supraclavicular Artery Island Flap Outcomes

**DOI:** 10.1002/oto2.175

**Published:** 2024-07-24

**Authors:** William Ruffin, Thomas J. Gal

**Affiliations:** ^1^ Department of Otolaryngology–Head and Neck Surgery University of Kentucky Lexington Kentucky USA

**Keywords:** head and neck reconstruction, pedicle flap, reconstructive surgery, supraclavicular flap, supraclavicular island flap

## Abstract

**Objective:**

Use of the supraclavicular artery island flap (SCAIF) in head and neck reconstruction has increased in recent years. Limited but improving experience among reconstructive surgeons with the procedure have exposed numerous issues associated with flap success. The objective of this study is to examine the role of flap size on viability.

**Study Design:**

Retrospective case series.

**Setting:**

Tertiary Academic Medical Center.

**Methods:**

Review of patients undergoing SCAIF reconstruction between January 2014 and March 2022 was performed. Flap failure was defined as >50% skin paddle loss. The total flap surface area was examined. Multivariable analysis was performed to evaluate the association of other variables associated with flap failure.

**Results:**

Eighty‐nine supraclavicular island flaps were reviewed. Mean patient age was 63.2 ± 11.4 years. Fifty‐five (61.2%) were male. Forty‐five flaps (50.6%) were used for the reconstruction of defects of the skin of the neck/face. Twenty‐nine flaps (32.6%) were utilized for defects of the pharynx/oropharynx, and 15 (16.9%) were utilized for oral cavity defects. Flap success rate was 94% (73/89). Flap site was not associated with flap failure (*P* = .46). Flaps >25 cm^2^ were 75% more likely to be successful. Multivariable logistic regression to assess the association of flap size in the context of other co‐morbidities indicated flaps >25 cm^2^ were 3.6 times more likely to succeed regardless of co‐morbidities, and patients with chronic obstructive pulmonary disease (COPD) have a 7‐fold risk of flap failure (odds ratio: 7.3, 1.72‐30.98, *P* = .007).

**Conclusion:**

An association with improved flap outcomes and larger skin paddles was observed in this series. The applicability of these observations to smaller flaps and larger series with more surgeons requires further study. Co‐morbidities, particularly, COPD, continue to impact flap outcomes.

Free tissue transfer remains the workhorse for complex reconstruction of the head and neck.^1^ For reasons of cost, time of procedure, and coexisting medical comorbidities, regional flap reconstruction has had somewhat of a resurgence as a reconstructive alternative for soft tissue defects of the head and neck.^1^, ^2^ This renewed enthusiasm is related to the reintroduction of flaps such as the supraclavicular island flap.^3^

The supraclavicular artery island flap (SCAIF) is a fascio‐cutaneous regional flap^1^ based off the supraclavicular branches of the transverse cervical artery with advantages of ease of harvest, good skin color match, and large skin paddle.^4^ It can be utilized for defects of the skin, oral cavity, and pharynx, with reported comparable qualities to the radial forearm flap.^5^, ^6^, ^7^, ^8^, ^9^, ^10^

Despite its advantages, universal adoption has been hindered by a lack of experience and training with the flap, as well as a notorious reputation for complications. In a survey of 221 American Head and Neck Society (AHNS) surgeons who reported performing supraclavicular flaps, just over half (53%) reported they were self‐taught the procedure.^3^ This would include the senior author. Additionally, of the surgeons not performing the flap, almost 10% stated they had either experienced bad outcomes with the flap or heard about bad outcomes from a colleague. Reports in the literature of high complications seemed to corroborate this, particularly, with mucosal site reconstruction.^11^, ^12^ However, other reports this decade have been more favorable.^13^, ^14^, ^15^, ^16^, ^17^, ^18^, ^19^, ^20^

After some initial success in patients who were poor candidates for a free flap, a more aggressive effort to incorporate the flap into our practice was initiated, and after navigating the learning curve and a making a few technical refinements, the overall number of successful flaps improved. Still, several complications continued that became more difficult to explain from a technical standpoint. The objective of this study is to review our experience with the SCAIF and variables that influence outcomes, particularly, flap inset site, size, and patient‐related factors that may impact flap success; thereby, influencing future patient selection. It cannot be emphasized enough, however, that this represents a self‐taught experience. Technical limitations, particularly over time, remain difficult to quantify.

## Methods

Demographic data collected included age, gender, diabetes mellitus, coronary artery disease (CAD), peripheral vascular disease (PVD), chronic obstructive pulmonary disease (COPD), and hypothyroidism. Charlson Comorbidity Index (CCI) was not utilized as it was felt to represent an aggregate of the very patient factors we were trying to individually identify. Furthermore, higher CCI, suggestive of more advanced medical disease, may be a contraindication to free tissue transfer. As a less extensive reconstructive option, the SAI cohort may be more biased to a population with greater co‐morbidities, but without introducing a free tissue group for comparison, given the previously acknowledged selection bias, this was omitted. Defect type was categorized as (1) “skin” for defects of the neck, face, or ear, (2) “pharynx” for defects of the hypopharynx, oropharynx, or neopharynx, or (3) “oral cavity” for defects of the tongue or floor of the mouth. Flap size was recorded as the area (cm^2^) of the harvested skin paddle and categorized for analysis. A history of prior head and neck radiation was recorded as a binary variable. Narrow field radiation to the larynx for early stage glottic cancer, however, was excluded. Smoking was classified in several ways for analysis. For the purposes of multivariate modeling, smoking was classified as a binary “never smoker” versus “ever smoker,” as well as a binary “current smoker” versus “former smoker/never smoker.” Given the paucity of “never smokers,” the latter classification was felt to be most useful. Smoking was also analyzed as a categorical variable, classifying patients as “current smoker,” former smoker (quit over 30 days ago), former smoker (quit over 1 year ago), and never smoker.

The primary outcome of interest was successful flap harvest and viability. Flap success/failure was coded as a binary variable. Flap failure was defined as complete necrosis of the skin flap, as well as any flap with a skin paddle loss of >50%. Equivocal flaps were, therefore, classified as flap failures. The vast majority of flaps, however, tended to either completely survive or completely fail. Partial loss of <20% was considered to be a flap that was successfully harvested from a technical standpoint. Demographics and clinical characteristics were reported as frequencies and percentages. Association of variables with flap failure were compared using Student's *t* test or Fisher's exact test, where appropriate. Univariable and multivariable logistic regression were performed to examine the association and interaction of variables with flap failure. All statistical analysis was performed using Stata 15.2 (StataCorp).

## Results

Between January 2014 and March of 2022, 89 supraclavicular island flaps were performed. Mean age of the patients was 63.2 ± 11.4 years. Fifty‐five of the 89 (61.2%) were male; 45 flaps (50.6%) were used for reconstruction of defects of the skin of the neck or face; 29 flaps (32.6%) were utilized to reconstruct defects of the pharynx or oropharynx, while the remaining 15 (16.9%) were utilized for oral cavity defects. Of the oral cavity defects, 3 used as rescue flaps. There were 2 fibula flaps where no perforators to the skin flap were identified, rendering it unusable. An additional flap was used for a lateral tongue defect after a failed radial forearm flap. Demographics of the patient cohort are listed in Table 1.

**Table 1:** Patient Demographics

	Success	Failure	*P* value
Age	63.56 ± 11.1	61.4 ± 13.4	.5
Gender			
Male	46	8	.5
Female	27	8	
Prior radiation			
Yes	38	3	.02
No	35	13	
Diabetes			
Yes	17	2	.6
No	56	14	
CAD			
Yes	12	10	.08
No	63	4	
PVD			
Yes	4	1	.08
No	69	15	
COPD			
Yes	14	9	.003
No	59	7	
Hypothyroidism			
Yes	11	4	.11
No	62	11	
Current smoking			
Yes	22	9	.06
No	51	7	
Reconstruction site			
Skin	39	6	.56
Pharynx	23	6	
Oral cavity	11	4	

Abbreviations: CAD, coronary artery disease; COPD, chronic obstructive pulmonary disease; PVD, peripheral vascular disease.

Overall flap success for the cohort, was 82% (73/89). Indicative of the learning curve involved with the development of the flap in our practice, flap success rate over the last 3 years was nearly 95%. Of the 16 flap failures, 10 were in current smokers (62.5%). All current smokers with flap failure had a history of COPD, compared with only 18.9% (14/74) in the flap success group (*P* = .001).

Other co‐morbidities, such as diabetes (*P* = .14), CAD (*P* = .22), PVD (*P* = .87), and hypothyroidism (*P* = .29) were not significantly associated with flap failure on univariable analysis using *χ* ^2^ testing. Prior radiation did reach statistical significance (*P* = .03). However, 36 (49.3%) of the 73 successful flaps had a history of prior radiation, compared to 3 of the 16 (18.8%) failed flaps. Thus, although there were a disproportionate number of patients with prior radiation across groups, it did not appear as if it were associated with flap failure and was not on multivariate analysis.

**Figure 1:**
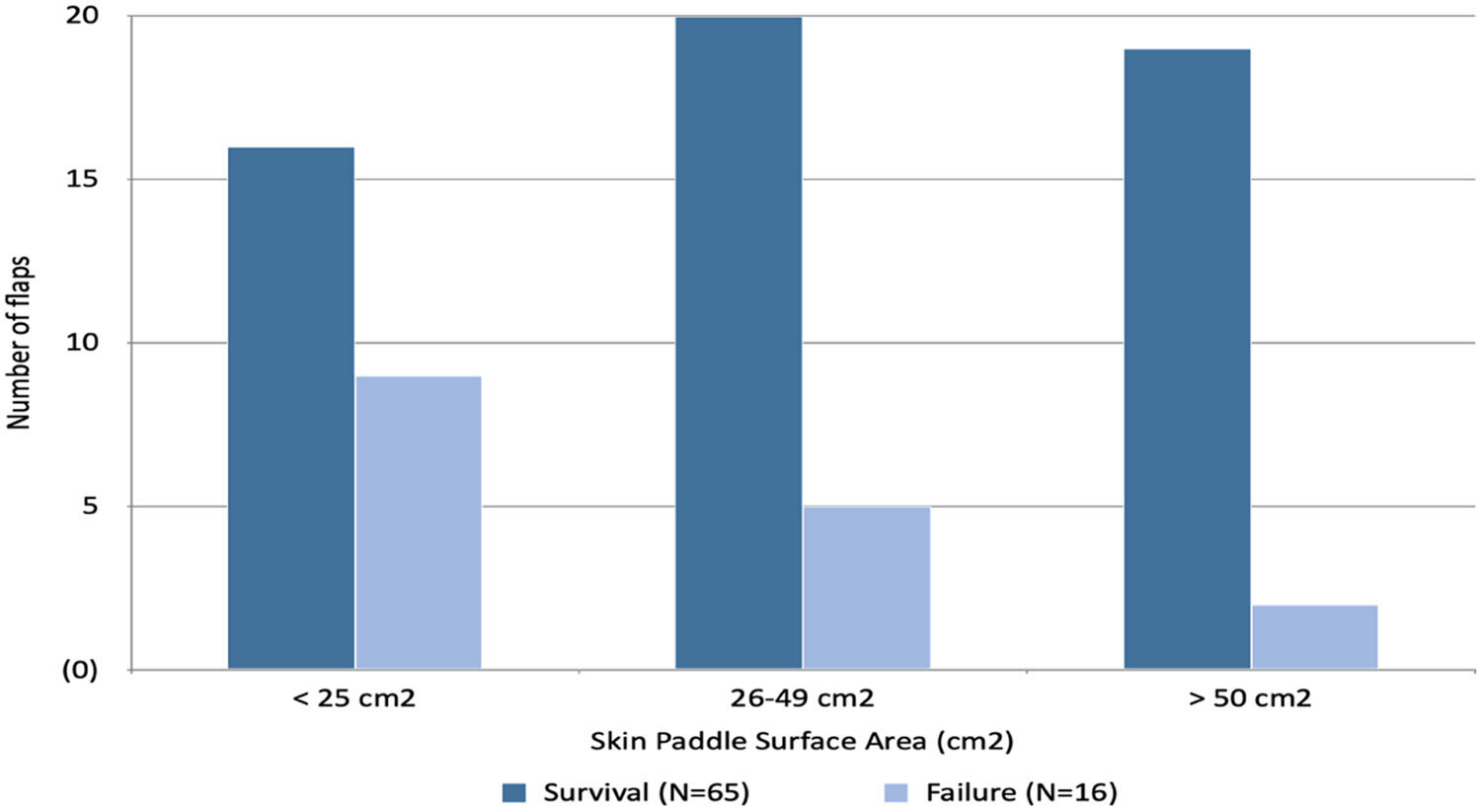
Impact of flap size on survival (*P* = .045).

Flap site (skin, pharynx, oral cavity) was not associated with flap failure (*P* = .46). Flap size was examined comparing surface area (cm^2^), and also categorized both as <25 cm^2^/26 to 49 cm^2^/and >50 cm^2^, as well as greater and less than 25 cm^2^. Failed flaps were found to have a smaller surface area, 32.3 ± 4.0 cm^2^, compared to successful flaps, 44.8 ± 3.1 cm^2^ (*P* = .059). The total surface area of 9 of the 16 flaps was less than 25 cm^2^, which was statistically significant regardless of how the flaps were categorized (*P* = .01, Figure 1). Flaps in the largest of the 3 flap categories were 81% more likely to be successful. Flaps were 75% more likely to be successful if greater than 25 cm^2^.

Multivariable logistic regression was then performed to assess the association of flap size in the context of other co‐morbidities, with flap failure. Variables included in the model included reconstruction site, flap size (< or >25 cm^2^), gender, prior radiation, smoking status, and COPD. After adjustment for other variables, flaps >25 cm^2^ were 3.6 times more likely to succeed. (odds ratio [OR] failure: 0.27, 0.065‐1.17). However, this did not reach statistical significance (*P* = .08). Patients with COPD were observed to have a 7‐fold risk of flap failure (OR: 7.3, 1.72‐30.98, *P* = .007). Current smoking, gender, prior radiation, and reconstruction site were not significantly associated with flap failure (Table 2).

**Table 2:** Multivariate Analysis of Factors Associated With Flap Failure

	Odds ratio	Standard error	*P* value	95% Confidence interval	
Gender	0.93	0.71	.9	0.21	4.17
Prior radiation	0.26	0.22	.11	0.05	1.38
Current smoking	1.46	1.13	.63	0.32	6.70
COPD	7.30	5.38	.007	1.72	30.98
Reconstruction site	1.29	1.14	.8	0.23	7.33
Flap size	0.27	0.20	.08	0.07	1.17

Abbreviation: COPD, chronic obstructive pulmonary disease.

## Discussion

The SCAIF has a long and complicated history. Medially based shoulder flaps in head and neck reconstruction were first described by Mütter in 1842.^8^, ^21^ In 1903, Toldt first described the arteria cervicalis superficialis vessel, originating from the thyrocervical trunk, exiting between the sternocleidomastoid and trapezius muscles.^22^ Based on these findings, various shoulder flaps of unpredictable vascularity were designed. The precursor to the modern supraclavicular artery flap was first described by Kazanjian and Converse in 1949.^7^, ^23^ The “in charretera” or acromial flap, as it was known, referred to the epaulet or ornamental strip of cloth on the shoulder of military uniforms. The flap was modified to an extent by Mathes and Vasconez in 1979, and referred to as the cervicohumeral flap.^24^ They described the vascular territory of the flap in formal anatomic studies. The flap fell out of favor, to some extent, after reports, most notably by Blevins and Luce, of a high incidence of distal necrosis.^11^ This was despite reports by Lamberty and Cormack, identifying the supraclavicular artery and demonstrating its reliability.^25^, ^26^ It was not until the late 1990s that Pallua rejuvenated interest in the flap, performing detailed anatomic studies defining the vascular patterns of what then became known as the “supraclavicular artery island flap.”^27^, ^28^ Use of the flap for head and neck reconstruction was first reported in 2000, and for oral cavity lining in 2005.^29^, ^30^ From there, numerous groups across the country began to report their successful experience with the flap for head and neck reconstruction.^5^, ^7^, ^8^, ^9^, ^15^, ^28^, ^31^, ^32^

The supraclavicular artery flap continues to be utilized relatively sparingly in head and neck reconstruction. In the previously mentioned survey by Day et al, only 53% of AHNS survey participants performed supraclavicular flaps.^3^ Of those who did, only 15.4% reported performing more than 30 in their career. This is in contrast to the countless hundreds of free flaps and other regional flaps such as the pectoralis flap that many senior head and neck reconstructive surgeons have performed over the years.

In this cohort of patients, flap selection was completely at the discretion of the senior author. Choice of flap was openly biased on the part of the author to make a conscious effort to incorporate the flap into our reconstructive armamentarium, and we recognize this as a limitation of the study. Given that the size and thickness were comparable to the radial forearm free flap, we hypothesized that the supraclavicular flap could provide a suitable alternative to the radial forearm. Similar arguments can be made for the submental island flap, which unquestionably provides a reliable regional reconstructive alternative to the radial forearm.^31^ Thus the most generalizable indication for SCAIF reconstruction over the study period would be defects that were otherwise amenable to radial forearm flap reconstruction that could not be otherwise reconstructed with a submental island flap. This would more precisely include (1) floor‐of‐mouth tumors with extensive Level I adenopathy which precluded submental flap harvest; (2) pharyngeal reconstruction after laryngectomy/laryngopharyngectomy; (3) facial, neck, and periauricular cutaneous defects larger than the submental island flap would accommodate; (4) for lateral defects, particularly, above the zygomatic arch. Tubed defects, or defects with complex geometry requiring significant tissue folding were preferentially not reconstructed with the SCAIF, and usually reconstructed with the forearm or anterolateral thigh flap. Cases managed by other members of the reconstructive team, which were not performing SCAIF flaps during the study period, were reconstructed by standard, usually microvascular techniques and were not included in the study.

Modifications that have been incorporated into the flap selection, design, and harvest include: (1) altered patient selection based on the results of this study, (2) movement of the skin paddle from the lateral deltoid to a more ventral location, (3) incorporating a wider soft tissue paddle extending from the deltopectoral groove anteriorly to the trapezius posteriorly which can be narrowed proximally toward the vascular pedicle to facilitate length and mobility, and (4) raising the flap as an axial flap, and then de‐epithelializing the proximal segment to create an island flap. The vasculature to the flap is immediately under the dermis.^4^ In order to effectively and efficiently raise skin flaps that are viable for donor site closure, dissection was often too deep, particularly, in thin individuals. The donor site is then widely undermined and usually easily closed primarily. Furthermore, (5) raising widely elevated skin flaps instead of a “tunnel” for inset, and always bringing the flap over the sternocleidomastoid instead of tunneling under it. Finally, in cases where perfusion was questionable with capillary refill or bright red bleeding after needle stick, (6) use of injectable fluorescent indocyanine green was utilized to assess flap viability prior to inset. In critically evaluating the first several years of this cohort, the flap success rate was 85.7%. In the most recent 3 years of the study, the success rate was 94.1%.

Benefits of the SCAIF include a relatively simple, efficient harvest, usually well under an hour. It has an extended arc of reach, and can easily reach above the zygoma, unlike the pectoralis or submental island flap. As such, it is also easily utilized for oral cavity reconstruction, including the palate. The skin is soft and pliable, very similar to the radial forearm. Unlike the forearm, there are no issues with exposed tendons at the donor site. Any wound breakdown at the donor site is easily managed with negative pressure wound therapy and/or skin grafting. Excessive folding or complex geometry, however, is not recommended by the senior author, and this is corroborated by the work of Kokot et al.^12^, ^17^

The association of COPD as a risk factor for flap ischemia in local and regional flaps is well established.^33^, ^34^, ^35^ The smoking prevalence in the head and neck population would certainly be a confounder in this population. Unexpectedly, however, after controlling for other variables, current smoking was not found to be statistically significant. The presence of COPD, however, was strongly associated with flap compromise in this cohort. Whether this is a function of the severity of the smoking history, or the overall tissue oxygenation in these presumably properly harvested regional flaps, is unclear.

In a critical appraisal of their experience with the SCAIF, Kokot et al, observed a higher rate of flap complications for mucosal defects relative to cutaneous reconstruction of the face, neck, and lateral skull base.^17^ In this cohort, the reconstruction site (mucosal vs skin) was not observed to be statistically significant (*P* = .45). In fact, the SCAIF was used as a rescue flap intraorally on several occasions, either in the setting of a fibula with no skin perforators or a failed forearm. If basic tenets of flap harvest are employed and the pedicle is not under tension, the recipient site would not appear to make a difference.

**Figure 2:**
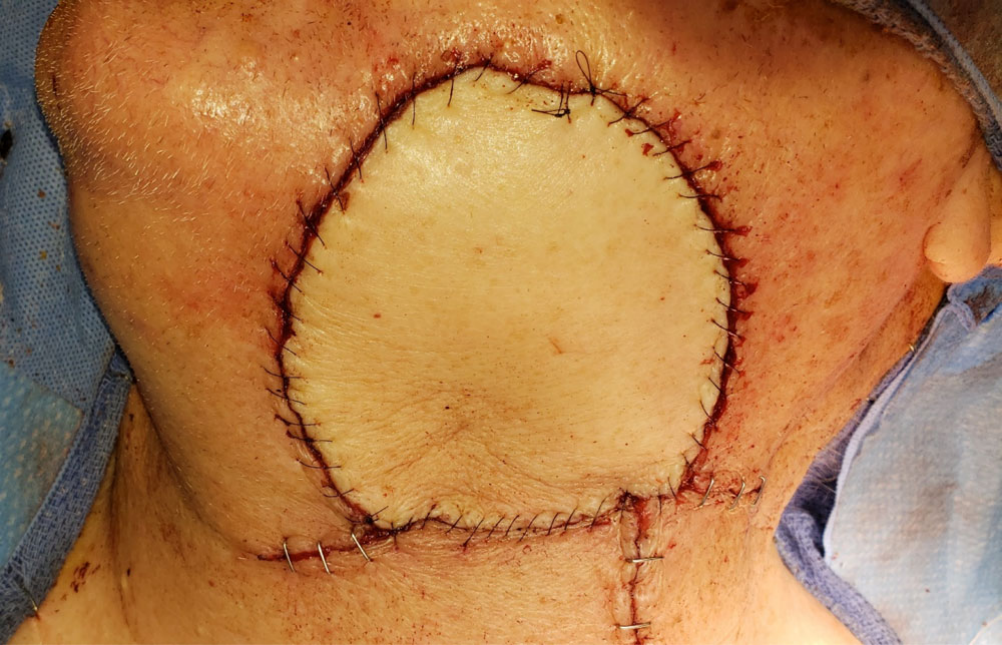
Supraclavicular artery island flap image.

Several studies have suggested that flaps with larger skin paddles are less likely to survive.^17^, ^36^, ^37^ In this study, it is apparent that flaps with surface area less than 32.3 cm^2^ were significantly more likely to fail, and those with surface areas greater than 44.8 cm^2^ were more likely to survive regardless of comorbidities. This seems somewhat paradoxical. One possibility is that a larger flap requires a greater vascular territory with increased vascular requirements. This would suggest that a smaller flap may do better. Alternatively, a larger flap may be more likely to incorporate the true vascular territory of the flap, ensuring flap viability. The experience of this cohort suggest the latter (Figure 2).

“An anatomically consistent random flap” appears to be the best description attributable to the SCAIF. The vascular territory of the supraclavicular artery, as well as contributions from the anterior supraclavicular artery perforator, appears to lie between the deltopectoral groove anteriorly, the trapezius posteriorly, crossing the acromion and extending on to the anterior face of the deltoid. Incorporating this soft tissue pedicle into all flaps has substantially improved success rates. This allows for harvest of a sizable skin paddle, up to 120 cm^2^. Conversely, raising a slightly wider subdermal pedicle in this manner has improved the success of smaller skin flaps in recent experience. Concerns for skin paddle viability prior to inset have been evaluated with indocyanine green angiography with promising results.^38^

A major limitation of this study is that, although association of medical co‐morbidities such as COPD with flap loss were observed, it is without question that a number of flap losses in this cohort were likely related to technical issues of the learning curve early in the experience. However, while experience improves with time, the presentation of patients with various flap defects and co‐morbidities, is much more random, making confounding unlikely. Other limitations include open bias in patient selection for SCAIF by the senior author, and external validity of results given the single surgeon experience.

## Conclusion

The observation in this series of improved flap success associated with larger SCAIF skin paddles may be nothing more than a function of a single surgeon experience. It may also be that a larger skin paddle more effectively includes the somewhat nebulous vascular territory of the flap. How to adapt these observations to the versatility, viability, and utility of smaller skin paddles requires further study, and likely additional surgeons. The observed association of medical co‐morbidities, particularly COPD, with flap outcomes, remain a well‐known nemesis in head and neck reconstruction.

## Author Contributions

**William Ruffin**, data acquisition, presentation, manuscript drafting; **Thomas J. Gal**, data acquisition, data analysis, manuscript drafting.

## Disclosures

### Competing interests

There are no financial interests or conflicts of interest to disclose.

### Funding source

None.
